# Compound 7 regulates microglia polarization and attenuates radiation-induced myelopathy via the Nrf2 signaling pathway in vivo and in vitro studies

**DOI:** 10.1186/s10020-024-00951-3

**Published:** 2024-11-04

**Authors:** Han Wu, Jianping Wu, Jianzhuo Jiang, Zeyu Qian, Shuang Yang, Yanze Sun, Hongxia Cui, Shengwen Li, Peng Zhang, Zhiqiang Zhou

**Affiliations:** 1 Department of Orthopedics, The Second Affiliated Hospital of Soochow University, Suzhou, China; 2 Department of Orthopedics, The Affiliated Yixing Hospital of Jiangsu University, Wuxi, China; 3 Clinical Research and Lab Center, Affiliated Kunshan Hospital of Jiangsu University, Kunshan, China; 4 Health Management Center, The Second Affiliated Hospital of Soochow University, Suzhou, China; 5 Department of Radiotherapy and Oncology, The Second Affiliated Hospital of Soochow University, Suzhou, China; 6 Department of Pathology, The Second Affiliated Hospital of Soochow University, Suzhou, China; 7 Department of Orthopedics, Haining People’s Hospital, Jiaxing, China

**Keywords:** Radiation-induced myelopathy, Compound 7, Nrf2, Polarization, Microglia

## Abstract

**Background:**

Radiation-induced myelopathy (RM) is a significant complication of radiotherapy with its mechanisms still not fully understood and lacking effective treatments. Compound 7 (C7) is a newly identified, potent, and selective inhibitor of the Keap1-Nrf2 interaction. This study aimed to explore the protective effects and mechanisms of C7 on RM in vitro and in vivo.

**Methods:**

Western blotting, quantitative real‐time polymerase chain reaction (qRT‐PCR), reactive oxygen species (ROS) and mitochondrial polarization, terminal deoxynucleotidyl transferase dUTP nick end labeling (TUNEL) assay, genetic editing techniques, locomotor functions, and tissue staining were employed to explore the protective effects and underlying mechanisms of C7 in radiation-induced primary rat microglia and BV2 cells, as well as RM rat models.

**Results:**

In this study, we found that C7 inhibited the production of pro-inflammation cytokines and oxidative stress induced by irradiation in vitro. Further, the data revealed that radiation worsened the locomotor functions in rats, and C7 significantly improved histological and functional recovery in RM rats. Mechanically, C7 activated Nrf2 signaling and promoted the microglia transformation from M1 to M2 phenotype.

**Conclusion:**

C7 could ameliorate RM by boosting Nrf2 signaling and promoting M2 phenotype microglia polarization in vitro and in vivo.

**Supplementary Information:**

The online version contains supplementary material available at 10.1186/s10020-024-00951-3.

## Introduction

Radiotherapy, also known as radiation therapy, is an essential form of cancer treatment and nearly 50% of cancer patients will receive radiotherapy at some point during their disease course (Leventhal and Young 2017; Jaffray and Gospodarowicz 2015). It utilizes focused ionizing radiation to kill cancer cells and shrink tumors to slow or stop their growth and spread. Ideally, radiotherapy targets only the cancer-affected area; however, it can also cause damage to healthy, cancer-free cells and surrounding organs. Radiation-induced myelopathy (RM) is a rare but serious spinal cord injury that can occur after radiotherapy for various malignant tumors in the neck, thorax, abdomen, or vertebral metastases (Schultheiss 2012; Okada and Okeda 2001). Clinically, the neurologic deficits of RM are typically progressive and irreversible, ranging from minor paresthesia to paralysis, seriously compromising the life quality of patients (Lee 2021).

The pathogenesis of RM involves complex processes, including direct neural damage, vascular injury leading to ischemia, and chronic inflammatory responses resulting in fibrosis and demyelination (Okada and Okeda 2001). The latency period between radiation exposure and symptom onset can vary from months to years, underscoring the complexity of its pathophysiology. Several factors contribute to the risk of RM, including the total radiation dose, fractionation schedule, and the volume of the spinal cord exposed. Typically, a total dose of 45–50 Gy is considered safe, but individual variability and adjunct therapies like chemotherapy can modify these thresholds (Jin et al. 2015). Given the limited treatment options for RM, novel preventative strategies are urgently needed (Ong et al. 2022; Carr et al. 2022). This study explores the potential of Compound 7 (C7) in modulating microglial response to mitigate RM via the Nrf2 signaling pathway.

Growing evidence has suggested that microglia, one of the major cell types stimulated in an irradiated central nervous system, have been shown to exert dual effects on neuroinflammation and neurogenesis based on their polarization phenotypes. Previous studies showed that amoeboid microglia were observed in the brain tissues of adult beagle dogs after irradiation (Nakagawa et al. 1996), and reactive microglia were detected in irradiated rat brains (Mildenberger et al. 1990). Besides, growing evidence also highlights that microglia exert dual effects on neuroinflammation and neurogenesis based on the polarization phenotypes (Fan et al. 2019; Kuboyama et al. 2021). The classical M1 phenotype secretes pro-inflammatory factors including tumor necrosis factor-α (TNF-α), interleukin-1 beta (IL-1β), and interleukin-6 (IL-6), inducing neuroinflammation and exacerbating neuronal injury. On the contrary, the alternative M2 phenotype releases anti-inflammatory mediators including transforming growth factor-β (TGF-β), IL-10, and IL-4, promoting neuroregeneration. Therefore, suppressing M1 polarization or promoting M2 microglia polarization might serve as a potential therapeutic strategy to improve the functional recovery of RM.

Studies have confirmed that nuclear transcription-related factor 2 (NF-E2 related factor 2, Nrf2) is a transcription factor that plays a key role in the oxidative stress response (Cuadrado et al. 2019; Zhou, et al. 2023). Under physiological conditions, Kelch-like ECH-associated protein 1 (Keap1) connects with Nrf2 in the cytosol for ubiquitin-mediated degradation; under the action of oxidants or electrophiles, Nrf2 dissociates from Keap1 and enters the nucleus, binding to the antioxidant response element (ARE) region of the downstream target genes. This initiates the transcription of various downstream detoxification enzymes and anti-oxidative stress genes, such as NQO1, HO1, and GCLC, and improves the ability of cells to resist oxidative stressed (Zhou et al. 2022; Zhang et al. 2023). In addition, studies have shown that the Nrf2 signaling pathway is also an important target for the prevention and treatment of radiation injury (Wang et al. 2019; Zheng et al. 2022) and the repair of spinal cord injury (Guo et al. 2022; Zhao et al. 2022; Zhang et al. 2022a). Thus, drug-targeted activation of the Nrf2 pathway is promising in ameliorating RM progression.

Recently, Jeffrey K. Kerns et al. have identified Compound 7 (C7), also known as KI-696, a highly potent and selective inhibitor of the Keap1-Nrf2 interaction (Davies et al. 2016), which can effectively boost the Nrf2 pathway in vitro and in vivo. The C7 demonstrates specific and robust affinity towards the Kelch domain, exhibits strong effectiveness in cellular assays, and effectively triggers the Nrf2 pathway in animal studies. Consequently, it serves as a thoroughly verified chemical probe for in vivo applications, offering a method to investigate the therapeutic possibilities of interfering with the Kelch/Nrf2 interaction. Thus, the purpose of the study is to investigate the protective effects of C7 on RM and reveal the underlying mechanisms. In this study, we first examined whether C7 could stimulate the Nrf2 cascade and suppress irradiation-induced cell death and apoptosis as well as inflammation and oxidative stress in vitro. Then, we proved that C7 could promote M2 polarization following irradiation. Further, we tested whether the irradiation-associated injury could be ameliorated by the Nrf2 pathway via C7 treatment. Finally, in vivo experiments were performed to demonstrate the effect and underlying mechanism of C7 on RM.

## Materials and methods

### Chemicals, reagents, and antibodies

**Fig. 1:**
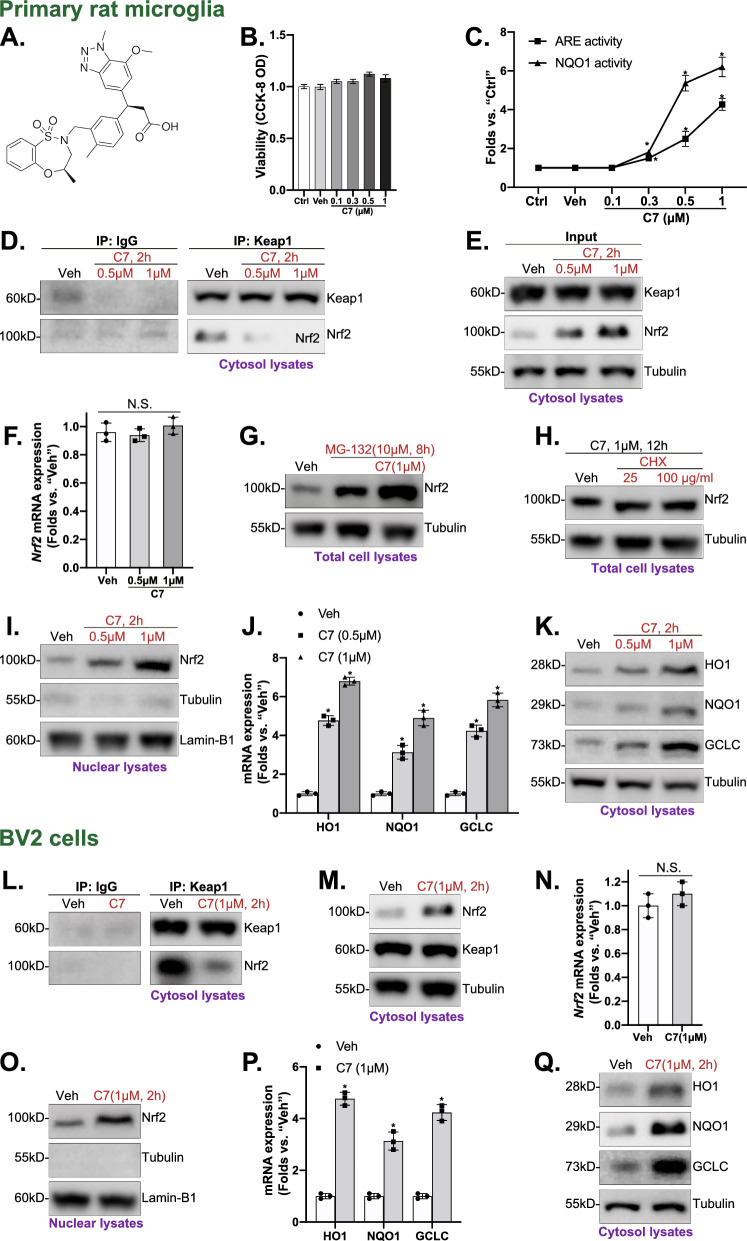
C7 activated Nrf2 cascade in microglia. Chemical structure of Compound 7 (C7) (**A**). After primary rat microglia (**B**–**K**) and BV2 cells (**L**–**Q**) were treated with C7, cell viability (CCK-8 OD) (**B**), as well as the relative antioxidant response element (ARE) activity and NQO1 activity (**C**) were examined; Keap1-Nrf2 association was tested via Co-Immunoprecipitation (Co-IP) (**D**, **L**); Proteins in cytosol and nuclear fraction lysates were examined by western blotting assays (**E**, **G**, **H**, **I**, **K**, **M**, **O**, **Q**), with mRNAs measured by qRT-PCR (**F**, **J**, **N**, **P**). The primary rat microglia were treated with MG-132 (10 μM) or plus C7 (1 μM) for 8 h, and total protein lysates were tested (**G**). The microglia were pretreated for 1 h with cycloheximide (CHX, 25 and 100 μg/mL), followed by C7 (1 μM) stimulation for another 8 h, and the listed proteins were shown (**H**). Expressions of the listed proteins were quantified and normalized to the loading control. Quantified values were mean ± standard deviation (SD, n = 3). “Veh” stands for vehicle control (0.1% DMSO). ^*^p < 0.05 vs. “Veh” cells. “N.S.” stands for the non-statistical difference (**F**, **N**)

C7 (purity > 98%, Fig. 1A) was provided by Shanghai Yuan Ye Biotechnology Co. Ltd. (Shanghai, China). The drugs were dissolved in 0.1% DMSO or normal saline. Cell Counting Kit-8 (CCK-8) was provided by Dojindo (Tokyo, Japan). Carboxy-2′,7′-dichlorodihydrofluorescein diacetate (carboxy-H2DCFDA) fluorescent dye was purchased from Invitrogen (CA, USA). Puromycin, polybrene, the Annexin V- fluorescent-activated cell sorting (FACS) assay kit, and cell culture reagents were purchased from Sigma-Aldrich (St. Louis, MO, USA). TRIzol reagent, Lipofectamine 2000, and other transfection reagents were purchased from Thermo Fisher and Invitrogen (Shanghai, China). The antibodies used in this study were demonstrated in Table 1. All reagents for cell culture were provided by Gibco (NY, USA). BV2 microglia cells were purchased from the Cell Bank of the Chinese Academy of Science.

**Table 1:** Information of antibodies

Antibody	Company	Concentration	Catalog Number	Application
Nrf2	Abcam	1:1000	ab62352	Western blotting
Keap1	Cell Signaling Technology	1:1000	8047	Western blotting
HO-1	Abcam	1:1000	ab13243	Western blotting
NQO1	Santa Cruz Biotechnology	1:1000	sc-32793	Western blotting
GCLC	Abcam	1:500	ab190685	Western blotting
Tubulin	Sigma-Aldrich	1:2000	T6199	Western blotting
Lamin B1	Abcam	1:1000	ab16048	Western blotting
Arg-1	Cell Signaling Technology	1:1000	93668	Western blotting
TNF-α	Abcam	1:1000	ab6671	Western blotting
iNOS	Cell Signaling Technology	1:1000	13120	Western blotting
β-actin	Sigma-Aldrich	1:5000	A5441	Western blotting
CD206	Santa Cruz Biotechnology	1:1000	sc-58986	Western blotting
Iba1	Wako Chemicals	1:1000	019–19741	Western blotting
HRP-conjugated goat anti-mouse IgG	Abcam	1:2000	ab6789	Western blotting
HRP-conjugated goat anti-rabbit IgG	Cell Signaling Technology	1:2000	7074	Western blotting
Iba1	Wako Chemicals	1:500	019‐19741	Immunofluorescence
iNOS	Cell Signaling Technology	1:200	13120	Immunofluorescence
Arg-1	Cell Signaling Technology	1:200	93668	Immunofluorescence
Nrf2	Abcam	1:200	ab62352	Immunofluorescence
Donkey anti‐rabbit IgG	Abcam	1:1000	ab150075	Immunofluorescence
Goat anti‐mouse IgG	Abcam	1:1000	ab150113	Immunofluorescence

### Primary rat microglia culture and irradiation

Microglia were isolated from 2 or 3-day-old neonatal Sprague–Dawley (SD) rats as described (Tamashiro et al. 2012). Briefly, the spinal cord was carefully dissected, mechanically triturated, and then digested with 0.25% trypsin/EDTA. Cells were cultured in DMEM/F12 with 10% fetal bovine serum (FBS). Microglia were obtained at about 10–14 days of culture by shaking the flasks and were verified by immunostaining of Iba1 (> 90% Iba1 +). Isolated microglia were cultured in DMEM/F12 with 10% FBS prior to indicated treatments. Microglia were divided into the control (Ctrl) group, irradiation (Rad) group, and C7 pretreatment for 24 h before the Rad (Rad + C7) group. Based on previous studies (Peng et al. 2014), cells in this research were irradiated with a single dose of 10 Gy at a dose rate of 2.0 Gy/min using a 4 MeV Electron Beam Linear Accelerator (Elekta Synergy, Sweden). The cell samples and culture supernatants were collected at 24 h post-radiation.

### Cell counting kit-8 (CCK-8) assay

Cell viability was assessed using the Cell Counting Kit-8 (CCK-8) assay (Dojindo, Japan). Cells were seeded in a 96-well plate at a density of 5 × 10^3^ cells per well and allowed to adhere overnight. Following treatment, 10 µL of CCK-8 solution was added to each well and incubated at 37 °C for 2 h. Absorbance was measured at 450 nm using a microplate reader. The relative cell viability was calculated by comparing the absorbance of treated cells to that of the control group.

### Western blotting

Cells and freshly extracted tissues from the injured spinal cord region were lysed in radioimmunoprecipitation assay (RIPA) lysis buffer containing phenylmethylsulfonyl fluoride (PMSF) and protease inhibitor (PI) for 10–30 min. Equal amounts of protein were separated by SDS-PAGE and then transferred to PVDF membranes (Millipore, USA). Membranes were blocked with 5% non-fat milk in TBST (Tris-buffered saline with 0.1% Tween-20) for 1 h at room temperature. After blocking, membranes were incubated overnight at 4 °C with primary antibodies against Nrf2, Keap1, HO-1, NQO1, GCLC, Tubulin, Lamin B1, Arg-1, TNF-α, iNOS, β-actin, CD206, and Iba1 (Table 1), followed by incubation for 1 h at room temperature with secondary antibodies for HRP-conjugated goat anti-mouse IgG, and HRP-conjugated goat anti-Rabbit IgG (Table 1). The membranes were washed three times and then developed using enhanced chemiluminescence (ECL) reagent (Bio-Rad), with ImageJ software (National Institutes of Health, Bethesda, MD, USA) used to quantify the band intensity.”

### Quantitative real‐time polymerase chain reaction (qRT‐PCR)

Total RNA was extracted from cells and freshly extracted tissues from the injured spinal cord region using TRIzol reagents (Thermo-Fisher Invitrogen, Shanghai, China) based on the manufacturer’s instructions. Reverse transcription was performed using a ReverTra Ace qPCR RT kit (Toyobo, Tokyo, Japan) and an ABI Prism 7600H fast Real-Time PCR system (Applied Biosystems, Foster City, CA). Melt curve analysis was performed to calculate the product melting temperature. Quantization of the listed mRNAs was carried out using the 2^−∆∆*C*t^ method. The primers are listed in Table 2, and *GAPDH* is used as the reference gene.

**Table 2:** Primer sequences used in qRT-PCR

Genes	Forward (5′-3′)	Reverse (5′-3′)
Nrf2	CACATCCAGTCAGAAACCAGTGG	GGAATGTCTGCGCCAAAAGCTG
HO1	CCAGGCAGAGAATGCTGAGTTC	AAGACTGGGCTCTCCTTGTTGC
NQO1	AGGCTGGTTTGAGCGAGTTC	ATTGAATTCGGGCGTCTGCTG
GCLC	GGAAGTGGATGTGGACACCAGA	GCTTGTAGTCAGGATGGTTTGCG
iNOS	TTGGCTCCAGCATGTACCCT	TCCTGCCCACTGAGTTCGTC
Arg-1	GGAACTCAACGGGAGGGTAAC	GAAGGCGTTTGCTTAGTTCTGTC

### shRNA

The Nrf2 shRNA lentiviral particles (sc-37030-V, Santa Cruz Biotech) or the Keap1 shRNA lentiviral particles (sc-43878-V, Santa Cruz Biotech) were individually added to cultured primary rat microglia for 24 h. To select stable cells, puromycin (5 μg/mL) was added to the complete medium for 10–12 days. Over 95% Nrf2/Keap1 knockdown in the stable cells was confirmed by western blotting and qPCR analyses.

### CRISPR-Cas9-mediated gene knockout (KO)

Cells were then transduced with lentivirus carrying CRISPR/Cas9-Keap1-KO puro-construct with Keap1-specific sgRNA (sc-400190-KO-2) and CRISPR/Cas9-Nrf2-KO puro-construct with Nrf2-specific sgRNA (sc-437374), provided by Santa Cruz Biotech. Then, following puromycin selection combined with fluorescence activated cell sorting (FACS)-mediated selection, stable cells formed Keap1 KO and Nrf2 KO were verified by western blotting and qRT-PCR analyses. The control cells were transfected with a lenti-CRISPR/Cas-9 empty vector with non-sense sgRNA (“Cas9-C”).

### Measurement of intracellular reactive oxygen species (ROS) and mitochondrial membrane potential

The carboxy-H2DCFDA dye assay was used to test the intracellular ROS levels based on the manufacturer’s protocol. In brief, with the according treatments, cells were incubated with 10 μM CM-H2DCF-DA for 30 min at room temperature. The fluorescence absorbance was measured by a fluorescent photometer (BD Biosciences, Shanghai, China) with excitation at 488 nm and emission at 525 nm, respectively. The mitochondrial membrane potential was examined by a JC-1 assay kit (Beyotime). The aggregate-to-monomer (red/green) fluorescence intensity ratio was used to quantify mitochondrial potential.

### Irradiation

Male adult SD rats (200–250 g) were purchased from Shanghai Animal Center of the Chinese Academy of Sciences and housed in a specific pathogen-free (SPF) facility. All animal experiments were conducted in accordance with the National Institutes of Health Guide for the Care and Use of Laboratory Animals and approved by the Institutional Animal Care and Use Committee of Soochow University. A total of 30 SD rats were randomly divided into 3 groups (n = 10 each): normal control (control) group, untreated irradiation (Rad) group, and radiation + C7 treatment (Rad + C7) group. Each rat was anesthetized via intraperitoneal injection of 3% sodium pentobarbiturate (45 mg/kg). The rat cervical spinal cords were irradiated using a 4 MeV Electron Beam Linear Accelerator (Elekta Synergy, Sweden). A 50 Gy radiation dose was strictly delivered to a 2 cm segment of the cervical spine field spanning C2-T2. The rats were observed daily for up to 5 months post-irradiation.

### Histological staining

Rats were deeply anesthetized with sodium pentobarbital (3%, 50 mg/kg) at indicated time periods after irradiation, followed by perfused intracardially with PBS. The injured spinal cords were dissected, fixed in 4% paraformaldehyde for 24 h, and embedded in paraffin. Samples were cut into 20 μm thick sections by a Leica slicer. Hematoxylin and Eosin (H&E) staining of spinal cord sections was performed based on the standard protocols.

### Immunofluorescence staining

The spinal cord sections were permeabilized in 0.5% Triton X-100 for 30 min, then blocked with 1% bovine serum albumin (BSA) for 1 h at room temperature, followed by incubation at 4 °C overnight with primary antibodies against Iba1, iNOS, Arg-1, and Nrf2 (Table 1). The specimens were incubated with the secondary antibody against donkey anti‐rabbit IgG and goat anti‐mouse IgG (Table 1) for 1 h at room temperature before nuclei staining with DAPI for 1 h at room temperature. The samples were triple-washed with PBS at each step. The images were captured by a fluorescence microscope (Leica, Wetzlar, Germany), and the relative fluorescence intensities were quantified by ImageJ software.

### Determination of the malondialdehyde (MDA) content and superoxide dismutase (SOD) activity

MDA levels were measured using the thiobarbituric acid reactive substances (TBARS) assay. Briefly, tissue or cell samples were homogenised in ice-cold phosphate buffer. The homogenate was mixed with thiobarbituric acid (TBA) reagent and incubated at 95 °C for 1 h. After cooling to room temperature, the samples were centrifuged and a supernatant was collected. The absorbance of the supernatant was measured at 532 nm using a spectrophotometer.

SOD activity was determined using a commercial SOD assay kit (Sigma-Aldrich). Samples were prepared by homogenising tissue or cells in cold lysis buffer, followed by centrifugation at 4 °C to collect the supernatant. The assay was performed according to the manufacturer’s instructions. In brief, the supernatant was mixed with the kit’s reaction buffer and the reaction initiated by adding the substrate. After incubation at room temperature, the absorbance was measured at 450 nm. Other procedures were carried out following the manufacturer’s protocols. Each experiment was conducted in triplicate.

### Terminal deoxynucleotidyl transferase dUTP nick end labeling (TUNEL) assay staining

TUNEL staining was performed to examine cell death of the injured spinal cord in accordance with the instructions of the TUNEL kit (Servicebio Fluorescein (FITC) TUNEL Cell apoptosis Detection Kit). In brief, sections were deparaffinized and rehydrated through a series of graded ethanol, followed by incubation in 20 μg/mL proteinase K for 20 min at room temperature. The samples were fixed in formaldehyde (4.0%, 5 min) after washing with PBS. After immersion in equilibration buffer for 20 min at room temperature, sections were incubated with a TdT enzyme reaction mixture for 1 h at room temperature and then incubated in the stop/wash buffer at 37 °C for 30 min to stop the reaction. DAPI was used to stain cell nuclei. Images were then captured by a fluorescence microscope (Leica). The average number of TUNEL-positive cells in 6 different fields per section was counted.

### Molecular docking analysis

The structure of C7 (PubChem CID 118170767) was obtained from the PubChem database (https://pubchem.ncbi.nlm.nih.gov/). The Keap1 protein (ID: 1X2J) was downloaded from PDB database (https://www.rcsb.org/) before being prepared for docking. Then, KI-696 was docked into 1X2J by Autodock (version 4.2.6). The default values are used for all parameters. Finally, the lowest energy conformations were chosen and visualized by virtue of PyMOL software (version 2.3.3) and Ligplot + (version 2.2.4).

### Behavioral assessment

Behavior analyses were performed for rats based on the Basso, Beattie, and Bresnahan (BBB) locomotor rating scale (Basso et al. 1995). Briefly, the rats were placed in an open field with a diameter of about 1.5 m and walked around freely for 3 min. Each rat was filmed and the locomotor functions of the hindlimb were scored by two independent observers blinded to the treatment regimen.

### Statistical analysis

Data were expressed as mean ± standard deviations (SD) of at least five independent experiments. Statistical analysis was performed using GraphPad Prism 8.4 (San Diego, CA, USA) software. Comparisons between two groups were evaluated by the two-tailed unpaired Student’s t-test, and multiple group comparisons were assessed by one-way analysis of variance (ANOVA) coupled with Tukey’s post-hoc tests. BBB scores were analyzed by the Bonferroni post hoc test. A *p* < 0.05 was considered statistically significant.

## Results

### 1. C7 activated Nrf2 cascade in microglia

The chemical structure of C7 was illustrated in Fig. 1A. To assess its impact on Nrf2 signaling, primary rat microglia were treated with varying concentrations of C7 (0.1–1 μM) for 8 h. CCK-8 assay revealed that C7 did not affect microglia viability (Fig. 1B), suggesting its safety. Additionally, C7 dose-dependently increased ARE and NQO1 activities (Fig. 1C), showing activation of Nrf2 signaling in microglia, with significant effects at 0.5 and 1 μM. Therefore, these concentrations were employed for further experiments. Next, the Co-Immunoprecipitation (Co-IP) was conducted and the results indicated that C7 (0.5 and 1 μM, 2 h) disrupted Keap1-Nrf2 association in microglia (Fig. 1D). Furthermore, C7 also elevated Nrf2 protein levels in the cytosol (Fig. 1E) and nucleus (Fig. 1I), while Keap1 protein (Fig. 1E) and Nrf2 mRNA levels (Fig. 1F) remained unchanged. Moreover, Nrf2 protein levels were unaffected by co-treatment with MG-132 (Fig. 1G) or cycloheximide (CHX) (Fig. 1H). Addtionally, C7 significantly upregulated mRNA and protein levels of Nrf2-ARE-dependent genes, including HO1, NQO1, and GCLC (Fig. 1J, K). These findings were corroborated by immunofluorescent staining (Fig. S1). Overall, C7 could disrupt Keap1-Nrf2 binding, promoting Nrf2 translocation to the nucleus and activating the Nrf2 signaling pathway in microglia.

In BV2 cells, the Keap1-Nrf2 connection was disrupted by C7 (1 μM) treatment (Fig. 1L), resulting in Nrf2 protein stabilizing in the cytosol (Fig. 1M). Keap1 protein (Fig. 1M) and Nrf2 mRNA (Fig. 1N) were not notably altered, while Nrf2 protein in nuclei (Fig. 1O), as well as mRNA (Fig. 1P) and protein (Fig. 1Q) levels of Nrf2-ARE-dependent genes were manifestly increased. Together, the results unveiled that C7 activated Nrf2 signaling in microglia.

### 2. C7 ameliorated irradiation-triggered cell death and apoptosis in microglia

**Fig. 2:**
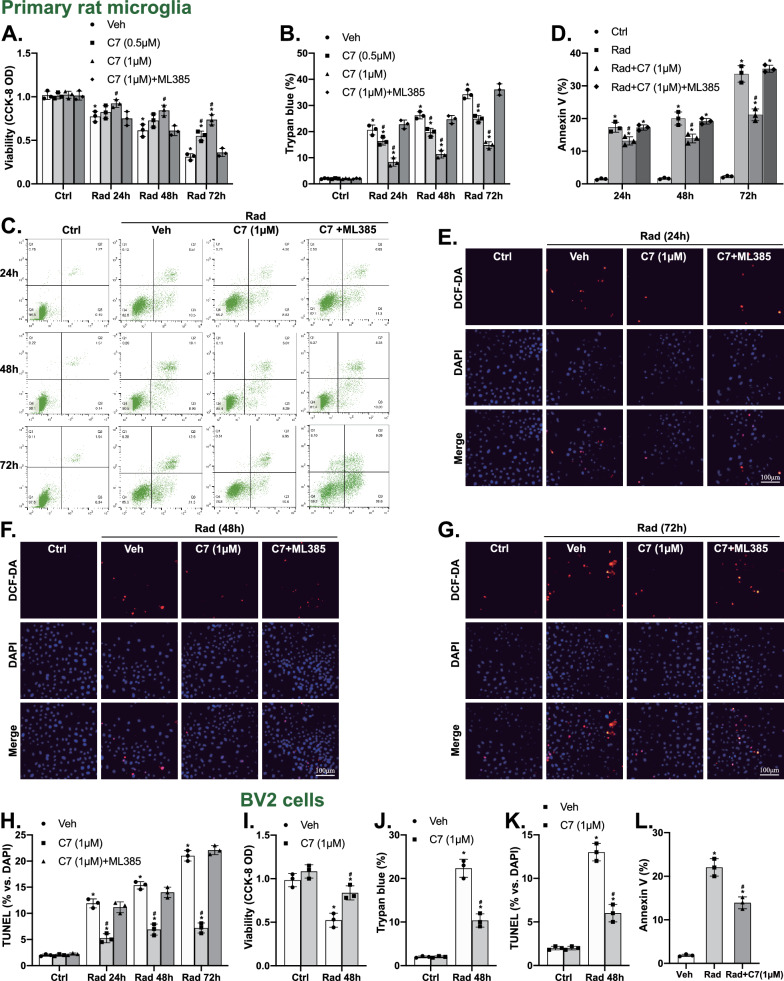
C7 ameliorated irradiation-triggered cell death and apoptosis in microglia. Primary rat microglia (**A**–**H**) and BV2 cells (**I**–**L**) were pretreated with the applied concentration of C7 or vehicle control (0.1% of DMSO, “Veh”), followed by ionizing radiation for indicated time periods (24, 48, 72 h). Cell viability was tested by CCK-8 (**A**, **I**) and cell apoptosis was investigated by Trypan blue (**B**, **J**), Annexin V flow cytometry (**C**, **D**, **L**), and nuclear TUNEL staining (**E**–**H**, **K**) assays. Quantified values were mean ± standard deviation (SD, n = 3). ^*^p < 0.05 vs. “Ctrl” cells. ^#^*p* < 0.05 vs. cells with irradiation but “Veh” pretreatment

Whether C7 could protect microglia from irradiation-stimulated cytotoxicity and apoptosis was examined. Iradiation (5 Gy) time-dependently (24, 48, 72 h) resulted in distinct cell viability reduction (CCK-8 OD, Fig. 2A) and cell death increase (Trypan blue, Fig. 2B) in primary rat microglia, which were manifestly attenuated by C7 (0.5, 1 μM) pretreatment. However, ML385, a known Nrf2 inhibitor, largely inhibited the cytoprotective effect of C7 (Fig. 2A, B). Furthermore, irradiation induced microglia apoptosis in a time-dependent manner, evidenced by increased Annexin V ratio (Fig. 2C, D) and TUNEL staining (Fig. 2E–H). Notably, C7 pretreatment inhibited radiation-triggered apoptosis activation, which was potently reversed by ML385 (Fig. 2C–H).

In BV2 cells, irradiation (5 Gy, 48 h) induced cell viability reduction (CCK-8 OD, Fig. 2I) and cell death (Trypan blue increase, Fig. 2J), as well as increased TUNEL staining (Fig. 2K) and Annexin V ratio (Fig. 2L), suggesting apoptosis activation. However, pretreatment with C7 (1 μM) potently inhibited these changes. The findings revealed that C7 pretreatment remarkably inhibited irradiation-induced cytotoxicity and apoptosis in microglia.

### 3. C7 inhibited irradiation-elicited oxidative stress injury

**Fig. 3:**
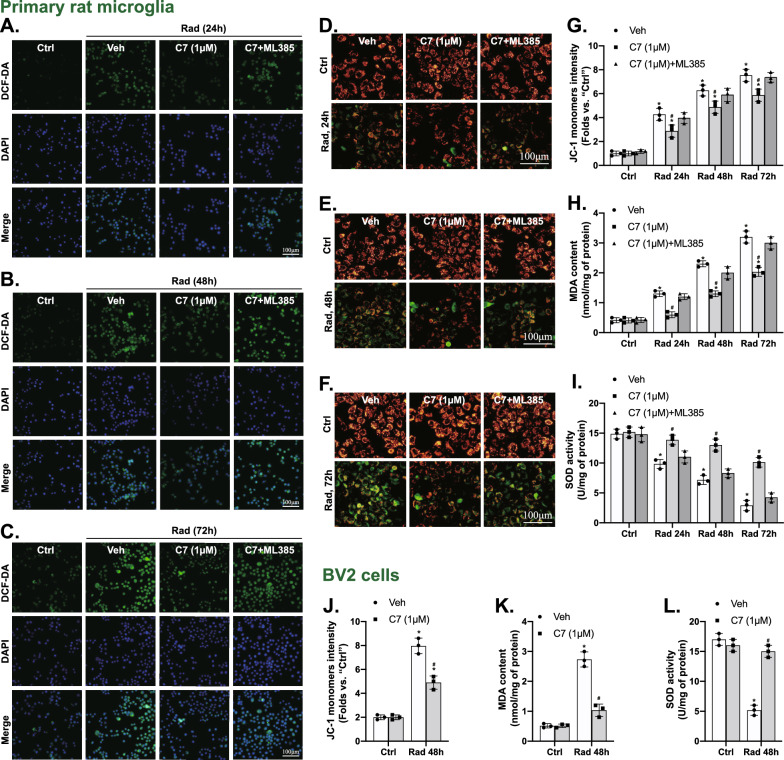
C7 inhibited irradiation-elicited oxidative stress injury. Primary rat microglia (**A**–**I**) and BV2 cells (**J**–**L**) were pretreated for 2 h with C7 (1 μM), or plus ML385 followed with or without irradiation. ROS production (**A**–**C**), mitochondrial depolarization (JC-1 staining, **D**–**G**, **J**), MDA content (**H**, **K**), and SOD activity (**I**, **L**) were measured, and results were quantified and normalized. Quantified values were mean ± standard deviation (SD, n = 3). ^*^p < 0.05 versus “Ctrl” cells. ^#^*p* < 0.05 versus cells with irradiation but “Veh” pretreatment. Scale bar = 100 μm (**A**–**F**)

The protective effect of C7 on irradiation-stimulated oxidative stress was then investigated. Primary rat microglia were treated with C7 (1 μM) and ML385 after ionizing radiation for different time points (24, 48, 72 h). As shown, irradiation elicited elevated ROS production (DCF fluorescent intensity, Fig. 3A–C, Fig. S2), mitochondrial depolarization (JC-1 green monomer fluorescence increase, Fig. 3D–G) and MDA content (Fig. 3H), as well as decreased SOD activity (Fig. 3I) in a time-dependent manner. Pretreatment with C7 (1 μM) potently reduced ROS production, mitochondrial depolarization and MDA content, while increased SOD activity, and the changes were blocked by ML385 treatment (Fig. 3A–I). Similar results for mitochondrial depolarization (Fig. 3J), MDA content (Fig. 3K) and SOD activity (Fig. 3L) were obtained in BV2 cells. These data suggested that C7 inhibited irradiation-induced oxidative stress in microglia.

### 4. C7 regulated microglia polarization following irradiation in vitro

**Fig. 4:**
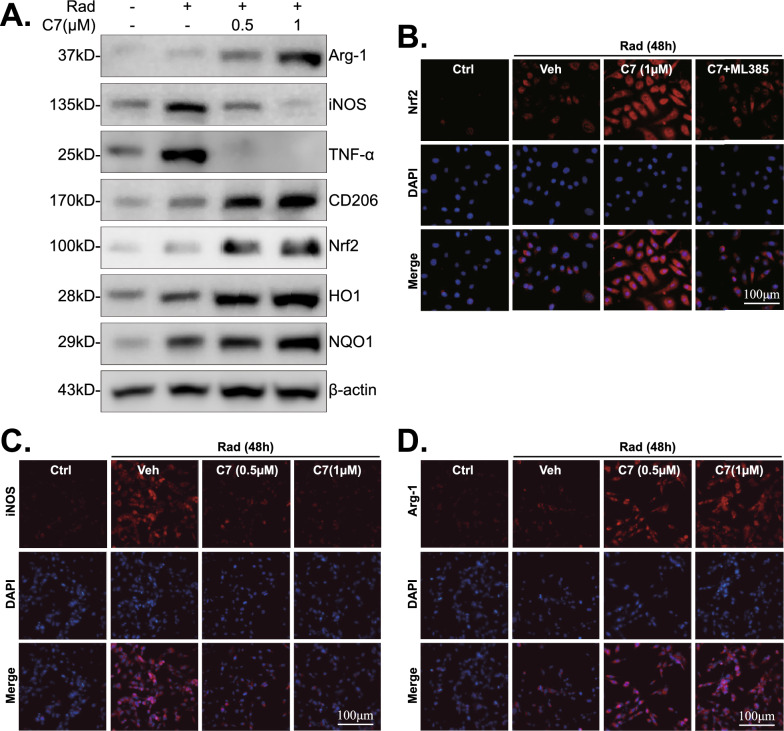
C7 regulated microglia polarization following irradiation in vitro. Primary rat microglia were pretreated with C7 (0.5 μM, 1 μM), followed by irradiation for 48 h, and expressions of listed proteins (**A**) were tested. Cell immunofluorescence of Nrf2 (**B**), iNOS (**C**), Arg-1 (**D**) were performed after irradiation (48 h). “Veh” stands for vehicle control (0.1% DMSO). Scale bar = 100 μm (**B**–**D**)

The effect of irradiation and C7 on microglia polarization and Nrf2 signaling was tested. Primary rat microglia were treated with or without C7 (0.5, 1 μM), followed by irradiation for 48 h. Our results implied that the protein expression of M1 phenotype microglia markers (iNOS, TNF-α) was remarkably elevated after ionizing radiation, while C7 (0.5, 1 μM) suppressed M1 marker expression and robustly promoted microglia M2 polarization, evidenced by the upregulated levels of M2 phenotype microglia markers (CD206, Arg-1) (Fig. 4A). Then, C7 (1 μM) administration led to a reduction in iNOS expression and an increase in Arg-1 expression. However, these changes were not statistically significant (Fig. S3). Therefore, we conclude that C7 alone does not significantly influence microglia polarization in the absence of external stimuli. What’s more, whether ko-iNOS suppress the effect of C7 against irradiation was investigated. As demonstrated in Fig. S4, ko-iNOS attenuated the protective effects of C7 against irradiation. This was evidenced by a reduction in cell viability (CCK-8 OD) (Fig. S4A), an increase in cell death (Trypan blue) (Fig. S4B), and enhanced mitochondrial depolarization (increased JC-1 green monomer fluorescence) (Fig. S4C, D) in primary rat microglia.

Besides, western blotting results manifested that C7 (0.5, 1 μM) treatment activated Nrf2 signaling pathway (Nrf2, HO1, NQO1) (Fig. 4A). Furthermore, the immunofluorescence results confirmed that Nrf2 was activated by C7 treatment, while ML385 abolished this (Fig. 4B, Fig. S2B). And the effect of C7 on microglia polarization was also examined by immunofluorescence of iNOS (Fig. 4C, Fig. S2C) and Arg-1 (Fig. 4D, Fig. S2D), which were similar to western blotting findings.

### 5. Nrf2 activation mediated C7-induced cytoprotection against irradiation in microglia

**Fig. 5:**
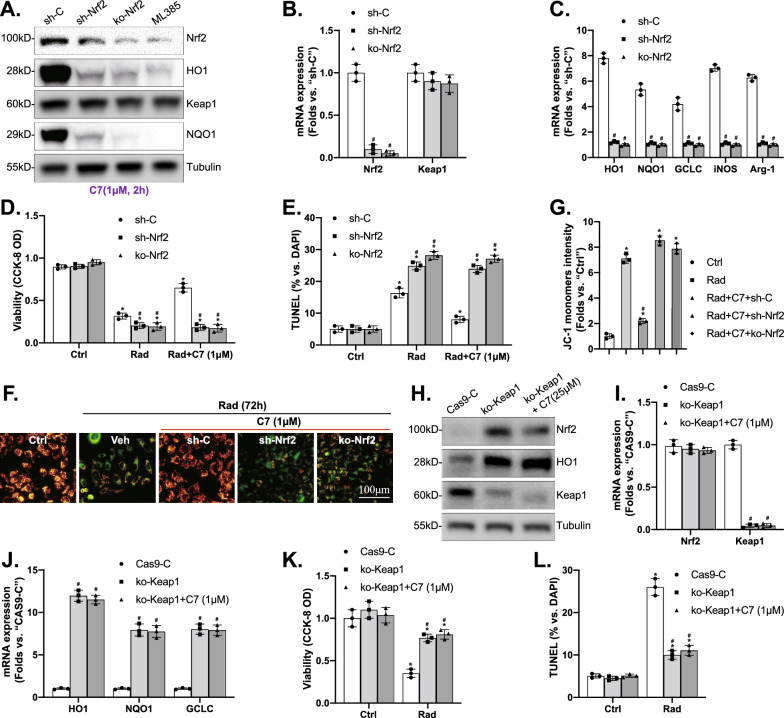
Nrf2 activation mediated C7-induced cytoprotection against irradiation in microglia. Stable primary rat microglia with Nrf2 shRNA (“sh-Nrf2”) and CRISPR/Cas9-Nrf2-KO construct (“ko-Nrf2”), as well as control cells with scramble control shRNA (sh-C) were established. The cells were treated with C7 (1 μM), and expressions of listed proteins (**A**) and mRNAs (**B**, **C**) were tested. Cell viability (CCK-8 OD, **D**, **K**), TUNEL (**E**, **L**), and mitochondrial polarization (**F**, **G**) were examined in Nrf2-silenced or ko-Nrf2 microglia. Alternatively, microglia transduced with CRISPR/Cas9-Keap1-KO-GFP construct (ko-Keap1) or with CRISPR/Cas9 empty vector (Cas9-C) were pretreated with C7 (1 μM), and expressions of listed proteins (**H**) and mRNAs (**I**, **J**) were tested. Expressions of the listed proteins were quantified and normalized to the loading control. Quantified values were mean ± standard deviation (SD, n = 3). ^*^p < 0.05 vs. “Ctrl” cells. ^#^*p* < 0.05 vs. “sh-C” or “Cas9-C” microglia

To test the link between Nrf2 activation and C7-induced cytoprotection against irradiation in microglia, genetic methods were utilized to silence Nrf2. Nrf2 shRNA lentivirus and stable microglia were established via puromycin selection and were transfected to primary rat microglia (sh-Nrf2). Besides, a CRISPR/Cas9-Nrf2-KO-GFP-puro construct was transduced to microglia to completely knock out Nrf2 (ko-Nrf2). Compared to the control microglia with scramble control shRNA (sh-C), C7 (1 μM)-promoted Nrf2, HO1, NQO1, GCLC protein, and mRNA expression were dramatically decreased in sh-Nrf2 and ko-Nrf2 microglia, while Keap1 remained unchanged (Fig. 5A–C), suggesting C7-promoted Nrf2 protein stabilization, as well as Nrf2-dependent gene expressions, were almost completely reversed after Nrf2 silence. Further, C7 (1 μM) was completely ineffective against irradiation-stimulated cytotoxicity and apoptosis in Nrf2-silenced or ko-Nrf2 microglia (Fig. 5D–G).

Then, microglia were transduced with CRISPR/Cas9-Keap1-KO-GFP construct (ko-Keap1) or with CRISPR/Cas9 empty vector (Cas9-C), and stable microglia were established. As shown, Keap1 depletion (Fig. 5H, I) did not affect Nrf2 mRNA levels (Fig. 5I), while motivated Nrf2 protein stabilization (Fig. 5H) as well as increased protein (Fig. 5H) and mRNA (Fig. 5J) expressions of HO1, NQO1 and, GCLC. What’s more, irradiation-induced viability reduction (Fig. 5K) and apoptosis (Fig. 5L) were largely inhibited by ko-Keap1. Notably, C7 (1 μM) failed to further increase Nrf2 activation (Fig. 5H, I), nor it could provide additional cytoprotection against irradiation (Fig. 5K, L). These results further supported that activation of the Nrf2 cascade is indispensable for C7-induced cytoprotection against irradiation in microglia.

### 6. C7 ameliorates irradiation-induced spinal cord injury via activating Nrf2 signaling and altering microglia polarization in vivo

**Fig. 6:**
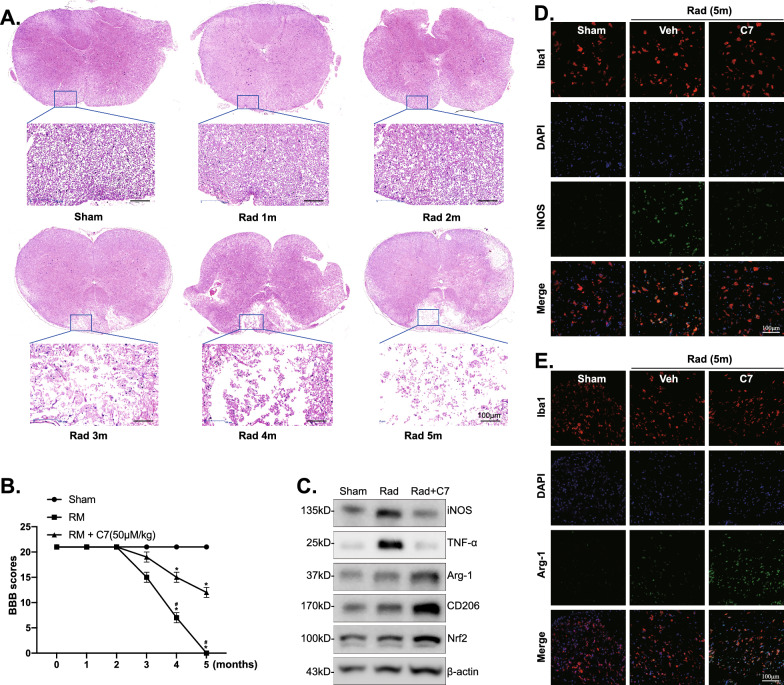
C7 ameliorates irradiation-induced spinal cord injury via activating Nrf2 signaling and altering microglia polarization in vivo. Histological analysis and behavior analyses in different experimental groups were evaluated by HE staining (**A**) and the Basso, Beattie, and Bresnahan (BBB) locomotor rating scale (**B**), respectively. The expressions of listed proteins of spinal cord tissues were tested by western blotting (**C**). The expressions of iNOS (**D**) and Arg-1 (**E**) in spinal cord tissues were examined by immunofluorescence. Quantified values were mean ± standard deviation (SD, n = 3). ^*^p < 0.05 vs. Sham group. ^#^*p* < 0.05 vs. RM group

To investigate the effect of C7 on RM in vivo, the RM rat model was established, and 50 mg/kg C7 once a day for 5 consecutive months was administered intraperitoneally in the Rad + C7 group. The degree of RM was evaluated by HE staining and BBB scores. As shown in HE staining (Fig. 6A), no histological evidence of demyelination was observed from the first 2 months after irradiation. However, a focally demyelinated zone in the dorsal funiculus developed from the third month. Consistent with the staining results, during a 5-month recovery period after RM, rats in the Rad + C7 group demonstrated potently higher BBB scores compared with the Rad group (Fig. 6B). Furthermore, the underlying mechanism of C7-triggered amelioration of RM was investigated. Expressions of M1-phenotype markers (iNOS, TNF-α), M2-phenotype markers (Arg-1, CD206) and Nrf2 were examined. Results of western blotting indicated that expressions of iNOS and TNF-α were increased after irradiation, which were remarkably ameliorated by C7 treatment, and expressions of Arg-1, CD206, Nrf2 were potently upregulated by C7 treatment compared with sham and irradiation groups (Fig. 6C). Furthermore, the representative M1-associated marker iNOS and M2-associated marker Arg-1 in injured spinal cord tissues were tested via immunofluorescence staining. As shown, the number of iNOS-positive microglia increased markedly after irradiation, and decreased potently after C7 treatment, while Arg-1-positive microglia increased dramatically after C7 treatment compared to sham and irradiation groups (Fig. 6D, E). Taken together, these results verified that C7 ameliorated RM via activating Nrf2 signaling and shifting microglia polarization from M1 to M2 phenotype.

### 7. Molecular Docking

**Fig. 7:**
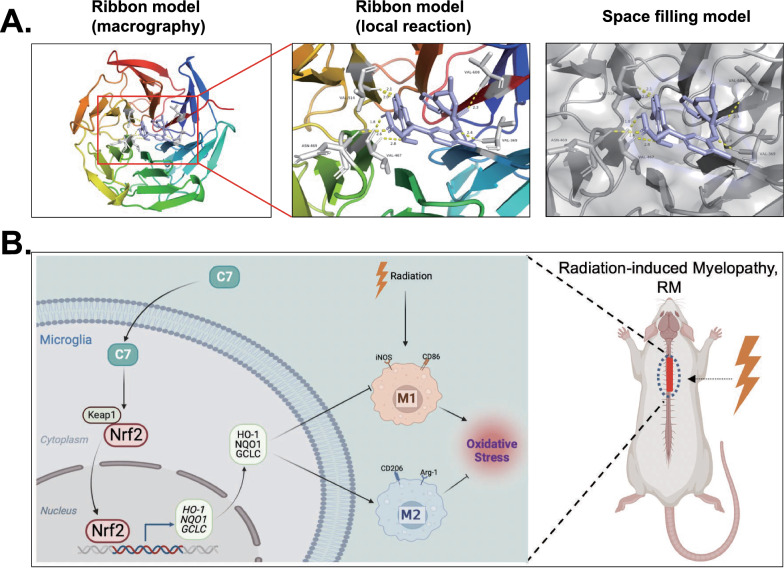
Molecular docking and the schematic mechanism of the protective effect of C7 on irradiation-induced microglia. C7 interacts with Keap1 in a docking study (**A**). A ribbon model is used to represent protein residues, with a two-dimensional binding model shown. C7 was able to dock strongly within the Keap1 binding site (affinity = − 7.34 kcal/mol). The binding of C7 in the Keap1 pocket is shown using a space filling model. Schematic mechanism showed that C7 protected microglia against irradiation-induced oxidative by activating Nrf2 and shifting microglia polarization from M1 to M2 phenotype (**B**)

To better understand the molecular mechanisms of C7-mediated Nrf2 activation, an in silico molecular docking study was performed with C7 on the structure of Keap1 Kelch domain to determine if C7 could interfere with the connection between Keap1 and Nrf2. The molecular docking of C7 with Keap1 protein (Fig. 7A, Fig. S5) revealed that C7 binds to Kelch domain of Keap1 protein efficiently with high-affinity (− 7.34 kcal/mol) hydrogen binding, with macro- and local-level views of these interactions shown using a ribbon model. Therefore, this suggested C7 may directly compete and restrict the binding of Nrf2 to Keap1 and thereby prevent its ubiquitination and subsequent degradation, leading to the activation of Nrf2. Further, the schematic mechanism of the protective effect of C7 on irradiation-induced microglia was depicted in Fig. 7B.

## Discussion

When the spinal cord is irradiated beyond the tolerable dose for the radiotherapy of tumors, a lesion may develop after a certain latent period, leading to spinal cord injury known as RM. However, there are few effective therapies could treat RM currently. The current study showed that C7 ameliorated RM by activating Nrf2 signaling and promoting M2 phenotype microglia polarization. First, we confirmed that C7 could interrupt the Keap1-Nrf2 interface and boost ARE-dependent genes in microglia. Next, we found that irradiation could induce microglia death and apoptosis, as well as increase oxidative stress injury, and these effects were abolished by C7 treatment. Furthermore, the irradiation protective mechanism of C7 was explored by investigating microglia polarization. The data indicated that M1 phenotype microglia were enhanced after ionizing radiation, while C7 treatment promoted M2 phenotype microglia polarization, supported by the alteration of polarization markers, such as decreased iNOS and TNF-α, as well as increased CD206 and Arg-1. In addition, we proved that Nrf2 activation mediated C7-induced cytoprotection against irradiation in microglia. Finally, in vivo results were also consistent with the in vitro findings. To sum up, C7 is a novel and effective compound that protects microglia against RM in vitro and in vivo.

Ionizing radiation exerts some biological effects by regulating ROS levels (Yuan et al. 2013). When cells are exposed to high doses of acute ionizing radiation, ROS level increases within mitochondria, leading to mitochondrial dysfunction. Then, damaged mitochondrial fragments generate and release more ROS within the cell, leading to programmed cell death (Averbeck and Rodriguez-Lafrasse 2021). Under normal conditions, ROS levels are maintained at low levels and are beneficial to normal cell metabolism. However, excessive ROS could cause an imbalance between ROS generation and scavenging, damage cells and tissues, and result in oxidative stress injury. Hence, it is evident that eliminating excessive ROS could protect cells from irradiation-induced injury. C7, as a Keap1 inhibitor, disrupts the Keap1-Nrf2 complex, thereby preventing the ubiquitination and degradation of Nrf2. The use of C7 is hypothesised to reduce intracellular ROS levels by upregulating the expression of Nrf2 target genes, which collectively contribute to the detoxification of reactive species and maintenance of redox homeostasis. Our current data confirmed that irradiation-induced increased cell death and apoptosis, and upregulated ROS production and mitochondria polarization in primary rat microglia, which were suppressed by C7 pretreatment, showing the evident ability of cytoprotection and ROS scavenging.

Morever, MDA is a cytotoxic product of lipid peroxidation and an indicator of free radical production and consequent tissue damage. The content of MDA can indirectly reflect the degree of cell oxidative damage (Chen, et al. 2018). In contrast, SOD is a metalloenzyme that catalyzes the dismutation of superoxide anion into oxygen and hydrogen peroxide (Miao and St Clair 2009). In this study, it was observed that the levels of ROS and MDA were increased and the activity of SOD was decreased significantly in irradiation-induced microglia, while C7 pretreatment alleviated this oxidative state. Hence, it is reasonable to hypothesize that by reducing the dose-limiting spinal cord toxicity, treatment of C7 might not only improve the life quality of patients but also allow the utilization of a higher dose regimen to improve the therapeutic effect of radiotherapy on tumors. The underlying mechanism needs further exploration.

One of the pivotal findings of this study is the influence of C7 on microglial polarization. Microglia, as the primary immune cells of the central nervous system, exhibit remarkable plasticity, which allows them to adopt different functional phenotypes in response to environmental cues. The shift from the pro-inflammatory M1 phenotype, characterized by the secretion of cytokines such as TNF-α and IL-1β, to the anti-inflammatory M2 phenotype, which secretes cytokines like IL-10 and TGF-β, is crucial for resolving inflammation and promoting tissue repair. This study demonstrates that C7 effectively suppresses M1 markers while enhancing M2 markers, thereby potentially reducing neuroinflammation and promoting neuroregeneration. This dual role of microglia in neuroinflammation and neurogenesis has been well-documented. Previous studies have shown that irradiation can lead to the activation of microglia and subsequent neuroinflammation (Liu, et al. 2022; Zhang et al. 2022b). Our findings extend this knowledge by showing that C7 can modulate this activation, shifting the balance towards a more reparative state.

Nrf2 is a member of the NF-E2 family of basic leucine zipper transcription factors encoded by the *NFE2L2* gene, regulating the antioxidant response that protects against oxidative damage. Previous studies have suggested that activating Nrf2 could prevent irradiation-induced brain (Liao et al. 2016), skin (Wang et al. 2019; Wei, et al. 2021), lung (Zheng et al. 2022; Dong et al. 2021) injury, and so on (Wakamori et al. 2022; Cameron et al. 2018). However, the role of Nrf2 in RM remains unknown. Additionally, accumulating evidence indicates that a large number of Nrf2 inducers have been discovered, most of which are electrophilic, and some are in clinical trials (Yagishita et al. 2020). Despite this, compounds currently in clinical trials lack selectivity due to their electrophilic mode of action and may cause pleiotropic effects. For example, well-studied Nrf2-activator CDDO analogs have been shown to respond to more than 500 molecules (Yore et al. 2011), and therapeutic outcomes of such compounds are likely to result from activities against multiple targets. Since these compounds are short of selectivity, their treatment effect may be ameliorated, and the results of activating Nrf2 will be poorly understood. On the contrary, non-covalent damage of the Keap1/Nrf2 connection may allow more specific regulation of this signaling pathway.

Our results revealed that the Nrf2 cascade was involved in RM and required for C7-induced cytoprotection against ionizing radiation in microglia. Specifically, drug inhibition of Nrf2 by ML385 and genetic elimination of Nrf2 worsened irradiation-induced microglia death and apoptosis and abolished the cytoprotective effect of C7, indicating that Nrf2 was necessary to mitigate the damage of radiotherapy. And we found that C7 had a strong ability of Nrf2 activation and neuronal protection against ionizing radiation in vitro and in vivo, resulting in disconnection of Keap1 and Nrf2, followed by Nrf2 activation, which was also verified by molecular docking method. In this study, the in vivo experiments corroborate the in vitro findings, indicating that C7 treatment improves both histological and functional outcomes in RM rat models. The significant improvements of BBB scores in C7-treated rats suggest its potential efficacy in clinical settings. These results are promising given the limited treatment options currently available for RM, a condition that severely impacts patients' quality of life. The clinical implications of these findings are substantial. Targeting the Nrf2 pathway with compounds like C7 could offer a novel therapeutic strategy for managing RM and potentially other radiation-induced injuries. The ability of C7 to promote neuroprotection and functional recovery by modulating microglial polarization and reducing oxidative stress highlights its therapeutic promise.

While our results are encouraging, it is crucial to acknowledge the limitations of our study. Firstly, it is essential to investigate the long-term effects of C7 treatment and its safety profile in more extensive and diverse animal models. Understanding the potential side effects and the optimal dosing regimen will be critical for translating these findings into clinical practice. Secondly, the transition from animal models to human treatment presents significant ethical and practical challenges. Invasive procedures, while feasible in animal studies, are often not possible in clinical settings due to ethical considerations. Therefore, while our findings contribute valuable insights into the pathophysiology of RM and potential treatment modalities, the direct applicability to patient care remains a complex and challenging frontier. Finally, further investigations into the molecular docking and binding interactions of C7 with Keap1 could facilitate the design of even more potent Nrf2 activators. The detailed understanding of these interactions will be crucial for optimizing C7 and developing next-generation compounds with improved efficacy and selectivity.

In conclusion, this research revealed C7 could ameliorate RM by boosting Nrf2 signaling and promoting M2 phenotype microglia polarization. The ROS generation will be suppressed by activating Nrf2 via C7 before irradiation exposure, consequently protecting microglia from death and apoptosis. To the best of our knowledge, we are the first to establish that C7 serves as a radioprotectant against RM. Due to its limited off-target effects, C7 may be a promising translational treatment to inhibit RM and might be beneficial to some cancer patients receiving radiotherapy. This study’s findings open pathways for clinical testing and potential reproduction in patient treatments. Our research demonstrates promising avenues in both in vivo and in vitro settings for mitigating the effects of RM. Future work should focus on translating these laboratory results into practical, ethical, and safe patient treatments, considering the challenges and limitations inherent in moving from animal models to human applications.

## Supplementary Information

Additional file 1.

## Data Availability

All data generated or analysed during this study are included in this published article.
