# Supplementary material for: Compound 7 regulates microglia polarization and attenuates radiation-induced myelopathy via the Nrf2 signaling pathway in vivo and in vitro studies
Source: Mol Med. 2024 Nov 4;30:198. doi: 10.1186/s10020-024-00951-3 (PMC11536861; doi:10.1186/s10020-024-00951-3)
Supplement: Supplementary file 1 — Additional file 1. [file 10020_2024_951_MOESM1_ESM.docx]

Supporting Information

**Compound 7 regulates microglia polarization and attenuates radiation-induced myelopathy via the Nrf2 signaling pathway *in vivo* and *in vitro***

**Han Wu^1†^, Jianping Wu^2†^, Jianzhuo Jiang^3†^, Zeyu Qian^1^,** **Shuang Yang^4^,** **Yanze Sun^5^, Hongxia Cui^6^,** **Shengwen Li^7^, Peng Zhang^1,4*^, Zhiqiang Zhou^1*^**

^1^Department of Orthopedics, The Second Affiliated Hospital of Soochow University, Suzhou, China.

^2^Department of Orthopedics, The Affiliated Yixing Hospital of Jiangsu University, Wuxi, China.

^3^Clinical Research and Lab Center, Affiliated Kunshan Hospital of Jiangsu University, Kunshan, China.

^4^Health Management Center, The Second Affiliated Hospital of Soochow University, Suzhou, China.

^5^Department of Radiotherapy and Oncology, The Second Affiliated Hospital of Soochow University, Suzhou, China.

^6^Department of Pathology, The Second Affiliated Hospital of Soochow University, Suzhou, China.

^7^Department of Orthopedics, Haining People’s Hospital, Jiaxing, China.

^†^Han Wu, Jianping Wu and Jianzhuo Jiang contributed equally to this work.

**^*^Correspondence:** Peng Zhang ([57994891@qq.com](mailto:57994891@qq.com)) and Zhiqiang Zhou ([zhouzhiqiang@suda.edu.cn](mailto:zhouzhiqiang@suda.edu.cn)).


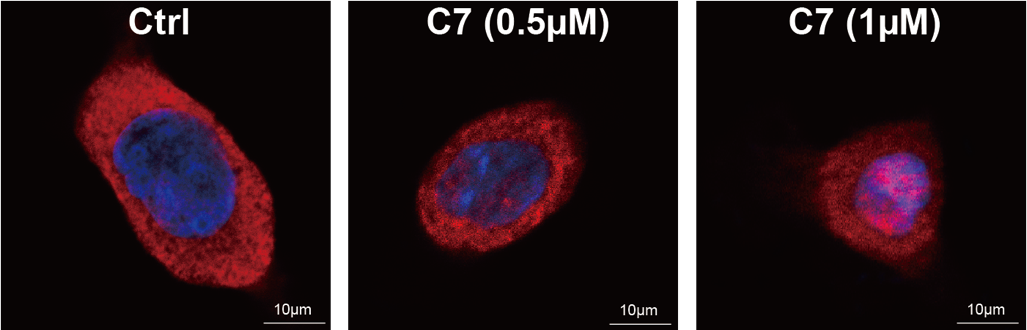


Figure S1. Nrf2 nuclear translocation was assessed via immunofluorescent staining in primary rat microglia, with 4′,6-diamidino-2-phenylindole (DAPI) used for nuclear detection (scale bar: 10 μm).


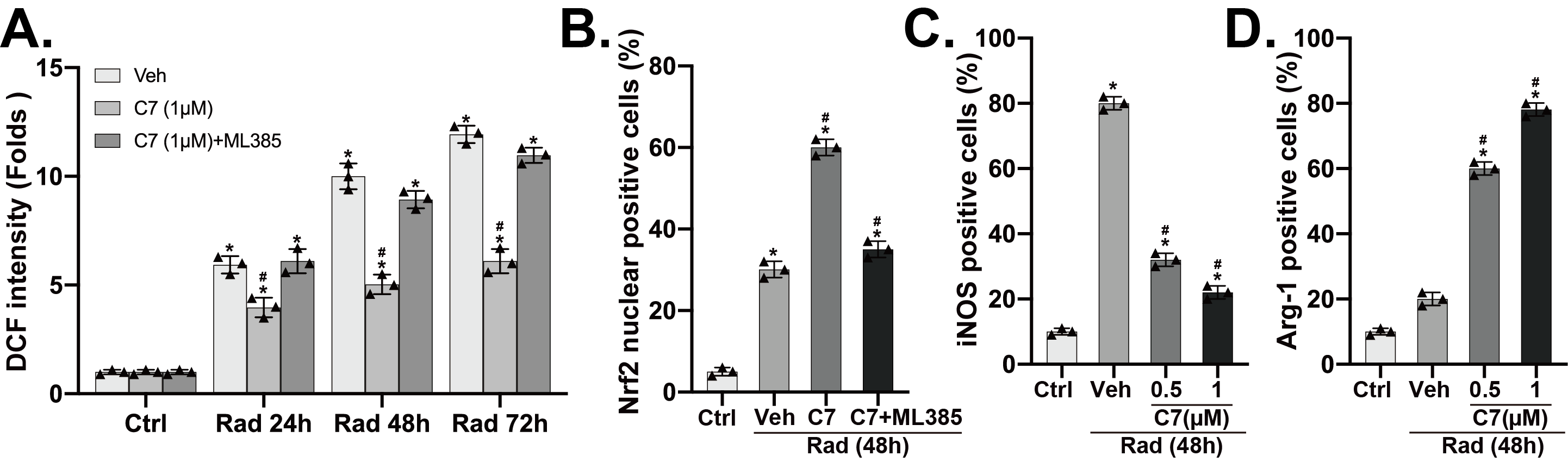


Figure S2. The statistical analysis for Fig. 3A-C (A), Fig. 4B (B), Fig. 4C (C) and Fig. 4D (D). Quantified values were mean ± standard deviation (SD, n = 3). ^*^*p* < 0.05 vs. “Ctrl” cells. ^#^*p* < 0.05 vs. cells with irradiation but “Veh” pretreatment.


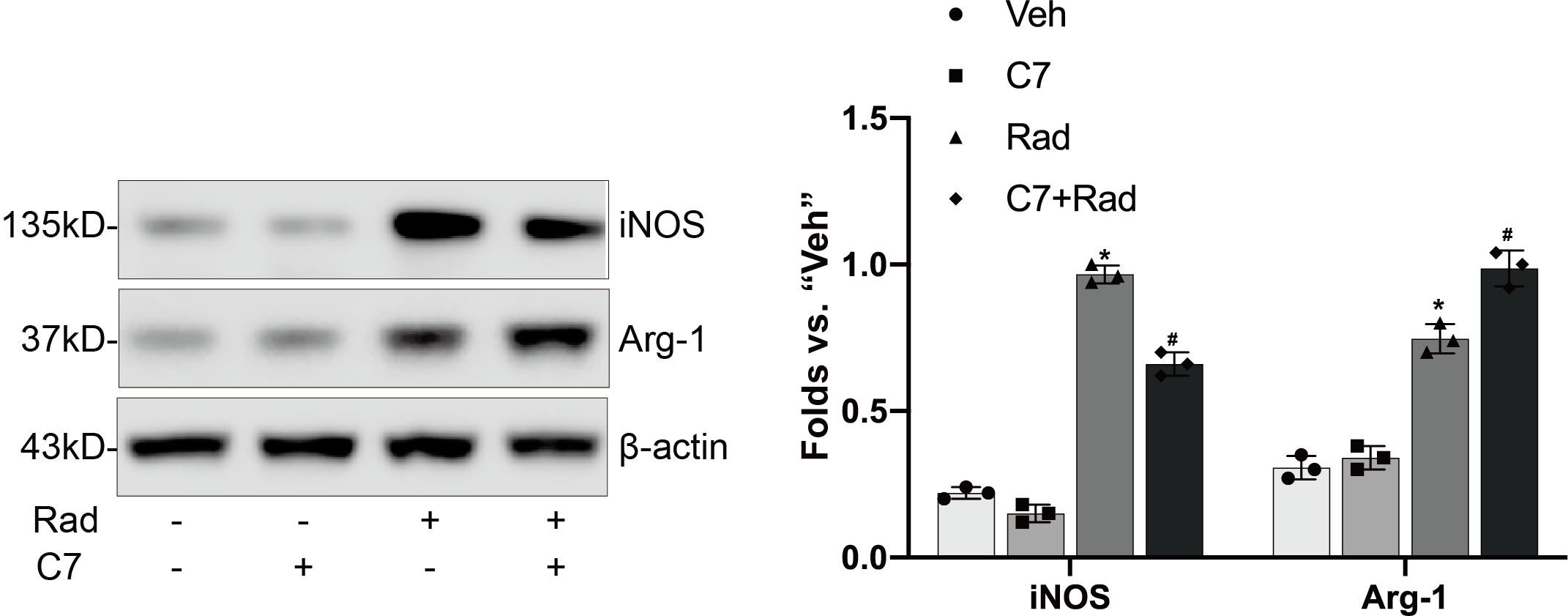


Figure S3. The effect of C7 (1 μM) on microglia polarization. Quantified values were mean ± standard deviation (SD, n = 3). ^*^*p* < 0.05 vs. “Ctrl” cells. ^#^*p* < 0.05 vs. cells with irradiation but “Veh” pretreatment.


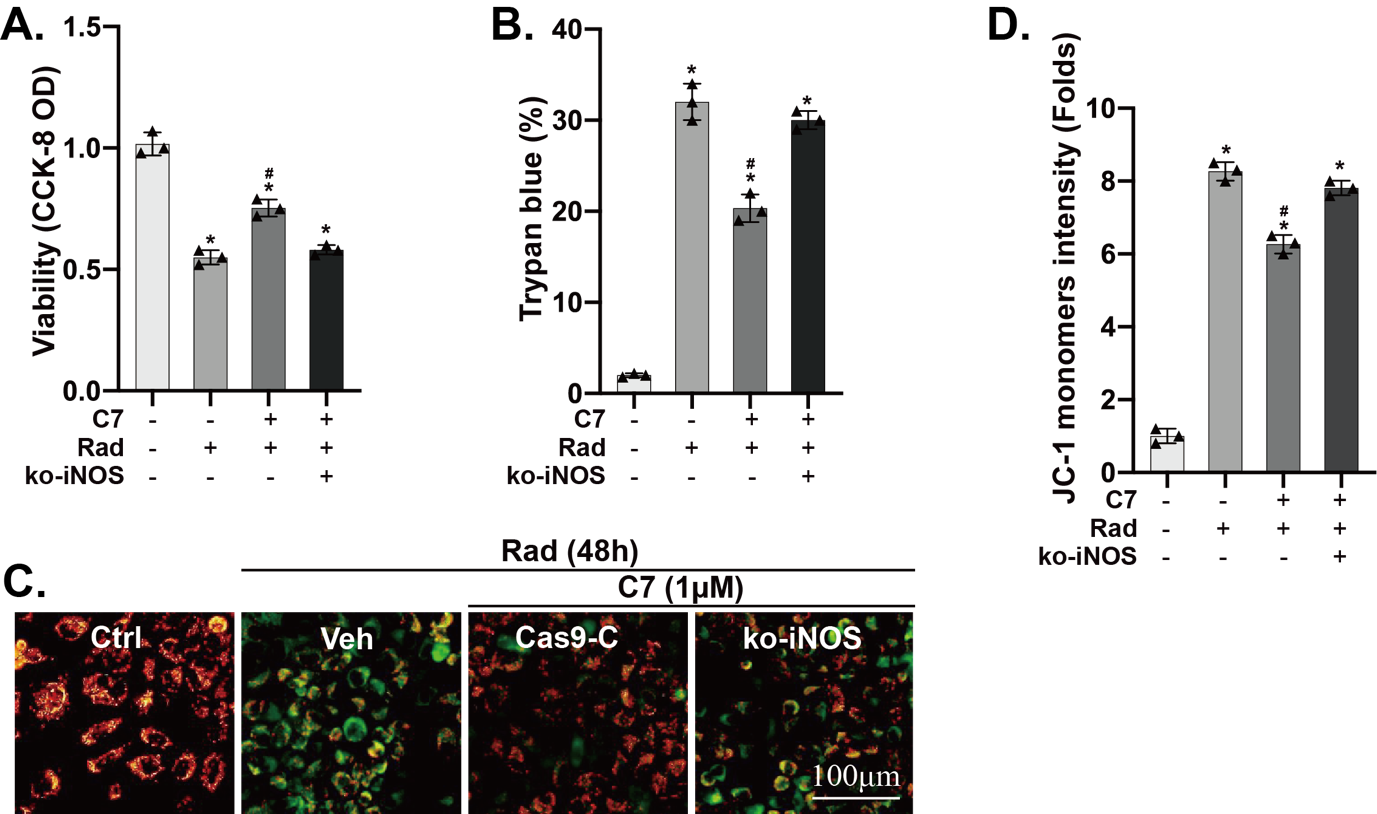


Figure S4. The effect of ko-iNOS on C7 effect against irradiation. ko-iNOS attenuated the protective effects of C7 against irradiation, as evidenced by a reduction in cell viability (CCK-8 OD) (A), an increase in cell death (Trypan blue) (B), and enhanced mitochondrial depolarization (increased JC-1 green monomer fluorescence) (C, D) in primary rat microglia. Quantified values were mean ± standard deviation (SD, n = 3). ^*^*p* < 0.05 vs. “Ctrl” cells. ^#^*p* < 0.05 versus cells with irradiation but “Veh” pretreatment.


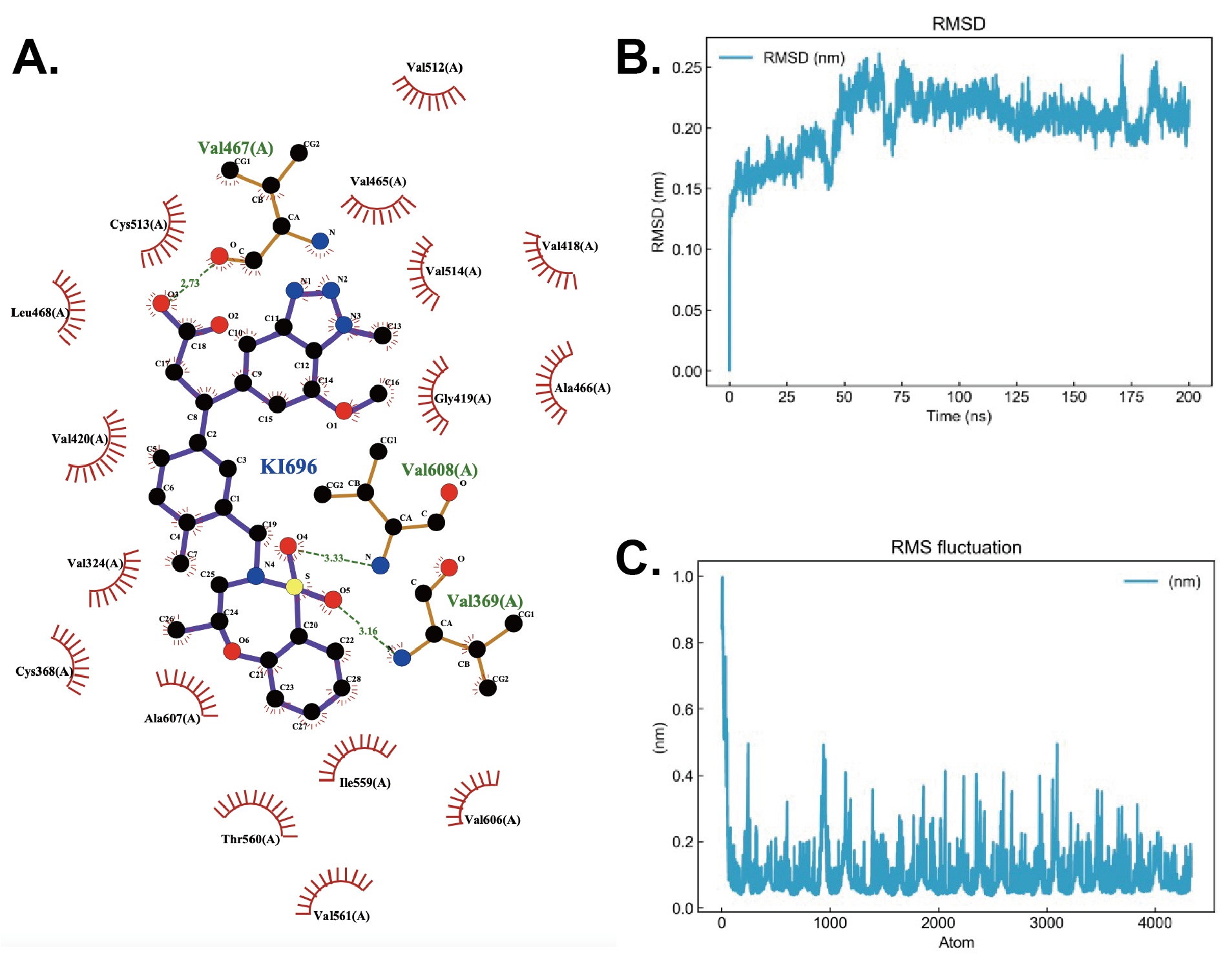


Figure S5. The 2D images of the docking results of KI696 and KEAP1 (A). The root mean square deviation (RMSD) of KI696-KEAP1 complex (B). RMSD is one of the most important factors to describe the stability of the MD simulation system. A lower RMSD value usually indicates better binding stability. The mean RMSD value of KI696-KEAP1 complex is approximately 0.20 and 0.26 nm. This demonstrates that KI696 and KEAP1 complex is stable in molecular dynamics simulations. The root mean square fluctuation (RMSF) of KI696-KEAP1 complex (C). RMSF is used to describe the average deviation between the displacement of each atom in a molecular system at a certain moment and its average position. In general, a small RMSF value indicates a stable molecular architecture. The key amino acid residues corresponding to KI696-KEAP1 complex showed limited fluctuations below 0.3 nm. This also demonstrates the stability of KI696 and KEAP1 complex.
